# Exploration the Supportive Needs and Coping Behaviors of Daughter and Daughter in-Law Caregivers of Stroke Survivors, Shiraz-Iran: A Qualitative Content Analysis

**Published:** 2015-07

**Authors:** Sakineh Gholamzadeh, Hamid Tengku Aizan, Farkhondeh Sharif, Basri Hamidon, Ibrahim Rahimah

**Affiliations:** 1 Community Based Psychiatric Care Research Center, Shiraz University of Medical Science, Shiraz, Iran;; 2 Department of Gerontology, University of Putra Malaysia (UPM), Malaysia;; 3 Department of Medicine, University of Putra Malaysia (UPM), Malaysia

**Keywords:** Aging, Family caregivers, Iran, Qualitative research, Stroke

## Abstract

**Background:**

The period of hospital stay and the first month after discharge have been found to be the most problematic stages for family caregivers of stroke survivors. It is just at home that patients and caregivers actually understand the whole consequences of the stroke. The adult offspring often have more different needs and concerns than spousal caregivers. However, relatively little attention has been paid to the needs of this particular group of caregivers. Therefore, this qualitative content analysis study aimed to explore the supportive needs and coping behaviors of daughter and daughter in-law caregivers (DILs) of stroke survivors one month after the patient’s discharge from the hospital in Shiraz, Southern of Iran.

**Methods:**

This is a qualitative content analysis study using semi-structured and in-depth interviews with a purposive sampling of seventeen daughter and daughter in-law caregivers.

**Results:**

The data revealed seven major themes including information and training, financial support, home health care assistance need, self-care support need, adjusting with the cultural obligation in providing care for a parent in-law, and need for improving quality of hospital care. Also, data from the interview showed that daughter and daughter in-law caregivers mostly used emotional-oriented coping strategies, specially religiosity, to cope with their needs and problems in their care-giving role.

**Conclusion:**

The results of this qualitative study revealed that family caregivers have several unmet needs in their care-giving role. By providing individualized information and support, we can prepare these family caregivers to better cope with the home care needs of stroke survivors and regain control over aspects of life.

## Introduction

Stroke is a complicated life-altering event for stroke survivors and their caregivers. Family caregivers feel a variety of unpreparedness and anxiety because of unexpected exposure to stroke and accepting a role as a caregiver.^1^ A large proportion of stroke patients who were discharged from hospital suffer from permanent disabilities, such as weakness, imbalance, mental changes, immobility and dependency in everyday activities.^2^ As the result of these functional impairments, stroke is more likely to affect most parts of their life, including the capacity to carry out activities of daily living (ADLs) as well as perform family and social roles.^3^ Therefore, they rely on their family members’ emotional, informational, and instrumental support for activities of daily living.^4^ After spouses, daughters and daughters in-law are more likely to assume the responsibilities of care for older persons.^5^ In Asian countries, about 39 to 77% of non-spousal caregivers are daughters in-law.^6^^,^^7^ Daughters or daughters-in-law are most likely to have conflicting requisitions between care-giving and their own social roles as a wife, mother, and employee.^8^^,^^9^ They have been labeled as the “patron”, “sandwich generation” or ‘‘women-in-the-middle’. These women are usually middle aged and have to manage their time and energy in several conflicting roles.^10^ Adult children, often have more different needs and concerns than spousal caregivers. Yet, their own needs often go unrecognized and, as a consequence, are not met. In addition, their informational needs have received less attention from the health care professionals in the health care systems.^11^ In addition, in a systemic review, Visser-Meily et al. (2005)^12^ revealed that most of studies were conducted on spouse caregivers and there is a gap in the literature on younger caregivers. Foster et al. (2001)^13^ in another systemic review demonstrated that the information needs of stroke patients and their caregivers were not being met and further studies were needed to determine specific educational needs of stroke survivors and their caregivers. To better support the family caregivers during the acute phase of stroke, we need to understand their experiences and care-giving needs. King and Semik (2006)^14^ indicated that caregivers face the most difficult time during the patient hospital stay and the first months following discharge. Therefore, since little data is available on the specific needs of this particular group of caregivers, this study aimed to explore the supportive needs, concerns and coping behaviors of daughter and daughter in-law caregivers (DILs) of stroke survivors one month after hospital discharge in Shiraz, Iran.

## Materials and Methods

*Research Design and Setting*

This is a qualitative study using content and thematic analysis to analyze the interview data. Content analysis is a method for subjective interpretation of content within text data by allowing the researcher to systematically code and identify themes and patterns.^15^ A purposive sampling of 17 caregivers included nine daughters and eight daughters-in-law took part in this exploratory qualitative study. Caregivers were recruited from two neurology departments of two large referral hospitals affiliated to Shiraz University of Medical Sciences (SUMS) in Iran. The caregiver had to be daughter and daughter in-law who had primary responsibility for caring the stroke survivors, lived in the same house with the survivors, and aged 18 years and over. The caregivers of stroke survivors with severe functional deficits were recruited because they experienced more problems during the acute phase of stroke recovery at home. To consider sampling with maximum diversity, we selected the participants from a broad range of daughter and daughter in-law caregivers with various characteristics (age, education, gender, socioeconomic status, etc.). The researchers attempted to interview with the well-informed caregivers who could provide extensive insight about the research questions.

*Data Collection*

Data were collected during seven months from July 2010 to February 2011. Seventeen daughters and DILs caregivers attended a focus group discussion (FGD) and individual in-depth interviews at the home of participants (for individual interview) and workplace of the researcher (in a nursing school for focused group). Each interview lasted from 60 to 75 minutes. The duration of conversation was flexible and based on the caregivers’ preference. Data were collected using a semi-structure interview consisting of open-ended questions. The interview began with general questions, such as “How do you perceive your needs as a stroke caregiver?” and moved to more specific, detailed questions as the interview advanced, such as “How do you feel your needs could better met?”, “How do you cope with the stress and demands of proving care for older stroke patients?”, “How do you think the health care system could help families in this situation?” The interviews were tape recorded with permission and subsequently transcribed. In addition, eight participants were interviewed twice to provide an opportunity to add the required data to and clarify what was said in the first interview. Sampling was continued until data saturation was achieved and no new categories could be identified from the analysis of the further interview and data.

*Data Analysis*

Content analysis with inductive coding was applied to examine the transcribed interview data. According to the content analysis process, the researcher transcribed the interviews and the transcriptions were read several times so that the researcher could immerse herself in the interview transcripts. All meaningful text units relevant to the study goal were determined and coded. Subsequently, open codes were identified, analyzed, compared, and grouped into categories.^16^Similar codes were grouped together based on a common characteristic. The purpose of grouping data was to decrease the number of categories by collapsing those that were similar or different into larger greater order categories.^17^^,^^18^ The data were analyzed by applying the constant comparison approach^19^which was used to compare and group together the different parts of data based on similarities and differences.

*Study Rigor*

For evaluating the rigor and trustworthiness of the research, a member check procedure was applied. For member-checking, four participants reviewed the transcription and emergent categories to understand whether the codes and categories revealed their needs and problems. In addition, the research team members reviewed the material (internal check) to evaluate the accuracy of the coding process and determine whether they applied similar codes and categories. Furthermore, a peer debriefing method was applied; in it, up-on completion of the content analysis, a random sample of three interviews, was assessed by two colleagues experienced in qualitative inquiry (external check). The results from open coding, grouping, and labeling were compared by the two reviewers and comments were applied to vertify the results.

*Ethical Considerations*

The research ethics committee of Shiraz University of Medical Sciences in Iran approved the study. All the participants were informed about the aims and contents of the research and signed the informed consent. They were also assured of anonymity and their right to leave the study at any time. 

## Results

Overall, 17 caregivers including nine daughters and eight daughters in-law (DIL) participated in the study. The participants’ ages ranged from 26 to 60 years, with a mean of 36.4 years. Table 1 shows the demographic characteristics of the sample.

**Table 1:** Demographic characteristics of daughter and daughter in-law caregivers

R_*_	**Age **	**Relation to stroke survivors**	**Marital Status **	**Number of ** **Children **	**Level of Education **	**Care Recipients **
P1	30	Daughter	Single	0	Diploma	Mother
P2	27	Daughter in-law	Married	1	Bachelor	Mother in-law
P3	40	Daughter in-law	Married	1	Diploma	Father in-law
P4	35	Daughter in-law	Married	2	Diploma	Father in-law
P5	43	Daughter	Married	3	Diploma	Mother
P6	29	Daughter	Married	1	Diploma	Father
P7	26	Daughter	Married	1	Diploma	Father
P8	35	Daughter in-law	Married	2	Diploma	Mother in-law
P9	45	Daughter in-law	Married	3	Elementary	Mother in-law
P10	33	Daughter in-law	Married	2	Diploma	Mother in-law
P11	60	Daughter	Widowed	4	Elementary	Mother
P12	29	Daughter in-law	Married	2	Diploma	Mother in-law
P13	42	Daughter	Married	3	Bachelor	Mother
P14	32	Daughter	Married	0	Diploma	Father
P15	40	Daughter in-law	Married	0	Diploma	Mother in-law
P16	35	Daughter	Single	0	Bachelors	Mother
P17	38	Daughter	Divorced	0	Tertiary	Mother

*Respondents

The data analysis revealed seven themes that provided a perspective on daughters and daughters in-law needs, including i) information and training, ii) financial support, iii) home health care assistance need, iv) self-care support, v) adjusting with the cultural obligation in providing care for a parent in-law, vi) need for improving quality of hospital care, and vii) needs for teaching problem-oriented coping approaches (Table 2). No obvious theme specific to the type of relationship emerged except cultural obligation in providing care for a parent in-law, which was discussed during the focus groups.

**Table 2:** Main categories and subcategories

**Caregivers’ Perceived Needs and Coping Behaviors**	
**Main Categories**	**Sub-categories**
Information and Training Needs	Skill Improvement: Practical training on core activities of daily living(ADLs)
	Knwoing: The knowledge to making decisions and taking appropriate action when faced with problems
Financial Support Needs	Home health care services expense
	Health supplies expense
	Lack of financial Afford
	Lack of social support
Home health care assistance need	Home health management
	Medical supplies
	Community based support
	Professional telephone advice
	Financial support through different payments
	Pharmaceutical assistance
Self-care support needs	Maintaining a balance between activity and rest
	Maintaining family function
	Maintenance of social function
	Maintenance of physical and emotional health
Needs for adjusting with cultural obligation in providing care for a parent in-law	Cultural duty of daughter in-law to care for husband parents
	Unequal division of caretaking duties
	Family Meeting
Need for improving quality of hospital care	Lack of proper communication from the health care professional
	Lack of preparedness and training prior to hospital discharge
	Dissatisfaction with hospital care
	Dissatisfaction with conventional medicine
Using Emotional oriented coping strategies / Needs for teaching problem-oriented coping approaches	Religiosity
	Positive Reappraisal
	Seeking emotional and social support
	Self-controlling
	Expression of emotions

*Information and Training Needs*

All family caregivers in this study presented a variety of educational needs related to the care for the stroke survivor with severe functional deficits. The most frequent and predominant educational need of family caregivers was managing the patient’s activity of daily livings (ADLs) and also patient’s moving and handling. One participant described her experience of the first month after discharge as follows: 

“*Last month, the patient was discharged; it was a difficult time for us. We had lots of problems. We did not have any knowledge in this field and we were not able to do our job efficiently. We did not know how to move her, how to clean her or how to put diaper on her. They didn’t instruct us at the hospital. They should give us a booklet to teach us how to take care of her, how to bath and how to help our patient to recover. We need to know how we can help our patients with peace and comfort for both of us.*” (P 8)

Caregivers expressed several educational needs about local resources: stroke risk factor and prevention strategies; sign and symptom of recurrent stroke; potential problems; hypertension; medications; physiotherapy outcome; frequency and necessity of the patient’s care; health education; and a place to call in emergency situations. Caregivers also lacked knowledge about the ways to prevent and manage post-stroke complications, such as muscle rigidity, pain, bed sore, bladder dysfunction, urinary tract infection, and bowel-related problems. A caregiver described her problem with managing the patient’s potential problems as follows: “*We find gradually that there would be a new obstacle ahead. You fix something but another thing cracks down*.”(P 3)

The other educational needs expressed by caregivers were managing the stroke survivor’s emotional and behavioral changes. In this sample, stroke survivors experienced a variety of behavioral changes, such as confusion, depression, personality changes, one side neglect, memory loss, insomnia, sense of humiliation and burden on family, and also speech problems. A participant explained her experience as follows:

“*Hospital should teach us how to behave in such situations with patients who are very sensitive and fragile. They get upset easily with simple things. They become different people after stroke. Their behavior, character and personality change .We should know how to treat them*.” (P 1)

Patients’ communication problems were also highly stressful for three caregivers (one daughter and two daughter in-law). 

*Financial Support Needs*

Low incomes, inadequate financial resources, and considerable out-of-pocket costs had created a sense of burden and frustration for 14 daughters and DILs caregivers. In this regard, a participant stated “*We really are under financial pressure. Stroke patient’s expenses are so high; it so expensive and difficult*.” (P17) The other source of financial distress was limited coverage of social health insurance. They also complained of high and different fees charged for the same type of home health care services in the private sectors.

*Home Health Care Assistance Need*

Lack of formal community support was also a major source of difficulty adding to the feelings of burden and distress in the family caregivers. Caregivers argued that the government should also provide financial assistance through different payments or at least by offering low interest loan, installing some medical equipment and devices such as bed, wheelchair, and suction machine. A participant stated “*We do not want money; they can give us some loans without interest, and it can be helpful and encourages us*” (P 17). The other issues suggested by the majority of caregivers (10 participants) were monthly home visit and professional telephone advice (13 participants). Holding a meeting with experienced caregivers so that new caregivers could benefit from their experiences and advice was also reported as being very important by two caregivers. Caregivers frequently reported the need for developing an ambulatory team or stroke support groups that help them to manage the patient at home.

*A Participant Stated*

“*Hospital should have ambulatory teams to take samples or help stroke patients with a bedsore at home. It would be great if they had such a team to take a blood sample and provide health care facilities at hospital rates. If they establish such ambulatory center soon, there will be no need to request assistance from private centers. It will be really helpful*” (P 5).

In addition, they demanded transportation services to be covered by the government or public hospital. “*It’s a big help if there is a place to call for help and transfer the paralyzed patients to a doctor’s office because we really have difficulty moving the patient*.”(P10) Other caregivers suggested that a stroke association should be established so that they can refer to and consult with physicians if their patient has problems.

*Self-Care Support Needs*

The other important need for the caregivers in the present study was need to self-care support. The theme of self-care support need is divided into four sub-themes including: *maintenance a balance between activity and rest, maintenance of family function, maintenance of social function, and maintenance of physical and emotional health.*

Nearly all caregivers experienced a sense of overwhelming with increased responsibilities such as the need to care for elderly parents/parents in-law along with caring for their own children and family, housekeeping, and arranging medical appointments. Also, multiple care-giving added extra distress for three caregivers. Multiple roles and their interaction with each other created role conflicts for 10 family caregivers. Caregivers were unable to meet the expectation of the other roles; therefore, they neglected their own health, family and particularly children’s needs as they focused mainly on caring for the stroke survivor. Inter-role conflict has led to major disruption of family function of the daughters and DILs caregivers as manifested in the following quotes:

“*We also do not live our own lives. We have had problems in our lives. We left our husbands and lives to take care of her. Well, on the one hand, we know we have responsibilities for our children, our husband, and ourselves and on the other hand we have to take care of her*“(P 8).

Also, most participants (13 caregivers) experienced conflict between care-giving tasks and their interpersonal and social life. In fact, the stroke survivor’s physical and emotional demands reduced the caregivers’ opportunity to socialize or be involved in social activities. Caregivers experienced a sense of confinement as they were unable to leave the patients due to a sense of commitment. A daughter in-law caregiver stated: 

“*A caregiver Confined to the home with the patient. They give up their plans. You cannot go and have a party. You cannot visit the family and friends. You feel responsibilities to care for patient as he may be alone at home and cannot go out*” (P 3).

Nearly all family caregivers experienced some physical and emotional distress, such as extreme tiredness, sleep disturbance, inadequate rest, physical pain (low back pain, headache, pain in shoulders, arms, and neck, and muscle stiffness), and other health problems. Predominant negative emotions that caregivers experienced included a sense of worn-out, breakdown, frustration, depression, nervousness, anxiety, anger, inability to concentrate, sense of loneliness, and helplessness. 

A daughter caregiver stated:

“*Now I am mentally broken down; sometimes I become nervous, scream, shout, and fight with everybody, old or young. I swear to God that at night I wake up by my heartbeats. I am worried about my children. I have lost my concentration. Sometimes I go shopping 2-3 times because I forget what I want to buy*”. (P12)

*Adjusting With the Cultural Obligation in Providing Care for a Parent In-Law*

Caregivers reported that in Iranian culture a son, predominantly the eldest son, is responsible to care for his older parents, which transformed into duties that had to be performed by his wife, or the daughter in-law. Consequently, daughter in-law caregivers experienced more suffering with a role that culture assumed for them. In this regard, a daughter in-law caregiver commented:

“*In some families, there is a belief that it is daughter-in-law’s duty to take care of her parents’ in-law. For example in our family, the daughter in-law is basically responsible. My mother in-law says that daughters cannot do anything without their husband’s permission. Since I am the bride of their son, so it is my main duty to take care of my father in-law. This idea is firmly established in families here*” (P 3).

According to the caregivers’ responses, holding family meeting would clarify the different responsibilities that could be shared with everyone who is able and willing to take on the responsibility. 

*Need for improving quality of hospital care*

This theme comprised four sub-themes, *lack of preparedness and training prior to hospital discharge, lack of proper communication from the health care professionals, dissatisfaction with hospital care, and dissatisfaction with conventional medicine*.

Lack of preparedness and training prior to hospital discharge was one of the main sources of caregivers’ dissatisfaction with the health care system. Caregivers felt that they lacked the knowledge and skill to care for the stroke survivor with severe functional deficits at home. Due to uncertainty and lack of competence, caregivers experienced a tremendous amount of emotional and physical exhaustion while providing care. Trouble in seeking information and lack of disclosure by health care providers were the most challenging aspect for family caregivers as manifested in the following statements of a participant: 

“*The most fundamental problem we had in the hospital was that the nurses and doctors hide the facts about patients’ condition from both the patient and family*”. (P 3)

Many caregivers reported that the doctors and nurses refused to communicate, answer their questions, and treat them with courtesy and respect.

“*I think lack of communication with patients and families is the major problem that hospitals have now. The hospital staff, especially physicians, should cooperate with the patient’s family more and should be in direct contact with them. However, it had many problems. I was really dissatisfied with them. When you ask a question, they do not answer. There are so many things and tips a nurse knows, which would take months for us to learn, but they did not tell us if we asked them*”. (P 5)

In addition, caregivers were dissatisfied with hospital care. The major causes of caregiver dissatisfaction with hospital care were health care providers’ lack of attention to the stroke patients’ emotional status, patient’s early discharge, lack of attention to the patient’s past medical history and pre-hospitalization medications, lack of continuity of care after discharge, and dissatisfaction with nursing care during the hospital stay. In this regard, a participant commented:

“*Doctors and nurses do not care about patient’s mental status but only care about patient’s physical health, such as doing CT scan and giving them their medicines. However, the main problem of the stroke patients with functional loss is their mental status. Someone who has been able to walk and do his affairs is now lying on bed just like a child. The patient gets hurt when I change his diapers and clean him. In my idea, the most important measure should be psychotherapy for both patient and family*”. (P3)

Family caregivers were also dissatisfied with conventional medicine. All eight caregivers in the focus group expressed their interest in herbal medicine rather than conventional medicines in terms of cost and effectiveness. Caregivers were dissatisfied with the effect of modern medicines on bedsore, muscle pain, rigidity, and digestion problems. A participant reported her experiences as follows:****

“*I used many ointments to treat my mother’s bedsore. Most of these ointments were expensive and ineffective. I referred to the herbal shop. He suggested a mixture. I applied the mixture to my mother’s bedsore every day. It cured her bedsore and there was no sign of redness*“(P 5).

Family caregivers requested the hospital to provide them with such information through written materials. Other issues discussed by family caregivers were related to the hot-cold food dichotomy and its scientific basis. The caregivers stated that some doctors and people believed in the hot and cold qualities of foods and advised stroke survivors to avoid eating foods with pure cold quality such as milk, yogurts, and fish. However, some doctors were not aware of such a dichotomy of foods. Therefore, caregivers perceived a sense of uncertainty about whether to follow this dietary pattern. They also requested that traditional medicine practitioners, in cooperation with conventional medicines, provide them with such information. 

*Caregivers Coping Behaviors*

Data collected from the interviews revealed that daughter and daughter in-law caregivers usually applied emotional-oriented coping strategies, especially religiosity, to cope with the stressful situation in their care-giving role. Nearly all of the family caregivers frequently stated that in a stressful situation they attempted to make a relationship with God through praying, reading Holy Koran, supplications, specially Ashura, seeking God support, put trust in God, and pleased with God’s will . In this regard, a participant commented: 

“*When I feel tired or upset, I read the Holy Koran and Ashura supplication and since I was doing it for God, I always said ‘Oh God! I am bearing these hard times for you. How tough the nights are and I am sleepless but I accepted with my heart and soul to do and bear these things because I am doing it for God and I am asking for his help. God knows I am not feeling angry and I do not expect anything from others. I always asked God to keep me in good health so that I can care for my father and except for His help not to let me seek others’ help*” (P 14).

The respondents in our study used several types of emotion-focused coping, the most predominant religious approach, and a combination of positive reappraisal (finding positive meaning through events), seeking emotional and social support, self-controll, and expression of emotions. 

## Discussion

The results of the present study showed that caregivers lacked knowledge and skills to manage physically dependent stroke patients and thus would like more information and hands-on training on managing the patients’ ADLs. Low, Payne, and Roderick (1999) found that caregivers’ ability to cope with stroke care will be improved with sufficient information on stroke.^20^ Without sufficient training, the caregiver may be exhausted physically and mentally and so unable to provide proper care. Nevertheless the information needs of caregivers are not being met across health care settings.^21^ Parallel with other studies^22-25^ managing stoke survivor’s cognitive and behavioral changes, disintegration in the family system and social life, lack of adequate time to fulfill all patients’ responsibilities, and caregivers’ physical and emotional exhaustion were the most challenges frequently reported by family caregivers during the first month after discharge. In addition, lack of formal home health care services and financial support were a source of stress for caregivers in this study. Moreover, in another study conducted in Iran, lack of financial and social supports was reported as the most significant issues that produced a feeling of incapacity and unhappiness.^26^ Lazarus and Folkman (1984) mentioned that social support is one significant source that may be accessible to caregivers as they appraise their conditions.^27^ Another contributor to stress for daughters and DILs caregivers was related to the cultural expectation that eldest son is primarily obligated to provide care for his old parents. In Iran aged care stem from a religious context. Islamic doctrine makes it obligatory for children to care for the aged parents. As such, with time it becomes part of the culture in Iran for children to take care of the aged parents. It becomes the responsibility of a good Moslem to ensure the elders have an honorable life.^28^

In addition, caregivers’ experiences revealed the need for improving quality of hospital care. All caregivers were dissatisfied with nurses/doctors communication, getting information, hospital care, and inadequacy of the information and training prior to discharge. Similarly, in a study conducted by Farahani, Sahragard, Carroll, and Mohammadi (2011) in Tehran-Iran, the problematic communication between the healthcare team, patients and their families was a major theme.^29^ Sirois (2008) in a systematic review reported that dissatisfaction with some aspects of conventional medicine, such as undesirable side effects, unsuccessful treatment, and inadequacy of doctor-patient relationship were the most common motivation factors stated by complementary alternative medicine (CAM) consumers.^30^ The finding also showed a similar pattern to the hot-cold dichotomy in the Chinese dietary and medical system. The equivalence between hot and cold is estimated necessary to physical well-being and those hot and cold qualities of foods and medicines should be noted in preserving an appropriate balance in curing sickness.^31^

In relation to the caregivers’ coping behaviors, the findings support other studies that reported family caregivers predominantly used emotional-oriented coping strategies to adapt with daily problems and stresses.^25^^,^^32^ Religiosity was a major way of coping for daughter and daughter in-laws caregivers. A connection with God perhaps affected the individuals’ appraisal of the situation, thereby assisting them to perceive the problems positively. It also gave purpose and hope to help the individual to adjust with the difficult events. In this way, the people will most probably use positive thinking when confronted with problems during the care of family members.^33^ In this sample, strategies which were most often used by caregivers to accomplish specific complicated tasks were emotional oriented coping approaches. Given that, caregivers experienced a wide variety of problems in their care giving role. Therefore teaching family caregivers how to solve these problems and to relieve their own stress is essential. However, coping process is affected by existing resources for coping which consists of skills and abilities, social resources, physical resources, tangible resources, and psychological resources.^34^ In this study, lack of financial, informational, and social supports was the most significant issue that produced a feeling of incapacity and lack of happiness in the caregivers. When there has been an appraisal that nothing can be made to alter harmful situation, caregivers have to manage the uncomfortable feelings through applying emotional coping approaches.

There are several limitations in the current study that must be acknowledged in future studies. This study evaluated the caregivers’ needs in the early post hospital period. Nevertheless, care-giving is a daily routine that changes in accordance with patient progression, such as the caregiver’s needs change. In addition, it is well-recognized that there are significant differences in the care-giving feeling and experience for spouses, adult children, and other sibling of stroke survivors which can limit application of the findings. Also, the small sample in this study is not necessarily representative of all Iranian caregivers. 

## Conclusion

The results of the analysis revealed that family caregivers had several unmet needs in the care-giving situation. Indeed, by recognizing the most frequent psycho-educational needs of caregivers and providing individualized information and support, we can prepare these individuals better to cope with home care needs of the person with stroke as well as their own physical, social, and personal needs. Moreover, caregivers applied predominantly emotion-oriented coping approach to adapt with the stressful situation, showing a need for teaching and introducing problem-oriented coping strategies in the training program. As a result, the study has important implications for health care system to recognize informal caregivers who are at higher risk of emotional distress and their potential needs, so that they can help them in the rehabilitation process. 

## References

[1] Greenwood N, Mackenzie A (2010). An exploratory study of anxiety in carers of stroke survivors. Journal of Clinical Nursing.

[2] Perry SB, Marchetti GF, Wagner S, Wilton W (2006). Predicting caregiver assistance required for sit-to-stand following rehabilitation for acute stroke. Journal of Neural Physical Therapy.

[3] Green TL, King KM (2009). Experiences of male patients and wife-caregivers in the first year post -discharge following minor stroke: A descriptive qualitative study. International Journal of Nursing Studies.

[4] Han B, Haley WE (1999). Family caregiving for patients with stroke: review and analysis. Stroke.

[5] Bee HL, Boyd DR (2003). Lifespan development.

[6] Kao HF, McHugh ML (2004). The role of caregiver gender and caregiver burden in nursing home placements for elderly Taiwanese survivors of stroke. Res Nurs Health.

[7] Kim JS (2001). Daughters-in-law in Korean caregiving families. Journal of Advanced Nursing.

[8] Barling J, MacEwen KE, Kelloway EK, Higginbottom SF (1994). Predictors and outcomes of elder-care-based interrole conflict. Psychology and Aging.

[9] Reid J, Hardy M (1999). Multiple roles and well-being among midlife women: Testing role strain and role enhancement theories. The Journals of Gerontology.

[10] Brody EM (2004). Women in the middle: Their parent care years.

[11] Richardson A, Plant H, Moore S (2007). Developing supportive care for family members of people with lung cancer: a feasibility study. Supportive Care in Cancer.

[12] Visser-Meily A, van Heugten C, Post M (2005). Intervention studies for caregivers of stroke survivors: a critical review. Patient Education and Counseling.

[13] Forster A, Smith J, Young J, Knapp P (2001). Information provision for stroke patients and their caregivers. The Cochrane Database Syst Rev.

[14] King RB, Semik PE (2006). Stroke caregiving: difficult times, resource use, and needs during the first 2 years. Journal of Gerontological Nursing.

[15] Hsieh HF, Shannon SE (2005). Three approaches to qualitative content analysis. Qualitative Health Research.

[16] Strauss A, Corbin J (1990). Basics of qualitative research: Grounded theory procedures and techniques.

[17] Dey I (1993). Qualitative data analysis: A user-friendly guide.

[18] Downe-Wamboldt B (1992). Content analysis: method, applications, and issues. Health Care for Women International.

[19] Strauss A, Corbin J (1998). Basics of qualitative research: Techniques and procedures for developing grounded theory.

[20] Low JT, Payne S, Roderick P (1999). The impact of stroke on informal carers: a literature review. Social Science & Medicine.

[21] O’Connell B, Baker L, Prosser A (2003). The educational needs of caregivers of stroke survivors in acute and community settings. J Neurosci Nurs.

[22] Bakas T, Austin JK, Okonkwo KF (2002). Needs, concerns, strategies, and advice of stroke caregivers the first 6 months after discharge. Journal of Neuroscience Nursing.

[23] Coombs UE (2007). Spousal caregiving for stroke survivors. Journal of Neuroscience Nursing.

[24] Grant JS, Glandon GL, Elliott TR (2004). Caregiving problems and feelings experienced by family caregivers of stroke survivors the first month after discharge. International Journal of Rehabilitation Research.

[25] Thomas M, Greenop K (2008). Caregiver experiences and perceptions of stroke. Health SA Gesondheid.

[26] Darani F, Riji HM, Abedi H (2010). How Iranian families response to the conditions affecting elderly primary health care. Research Journal of Biological Sciences.

[27] Lazarus RS, Folkman S (1984). Stress, appraisal, and coping.

[28] Aghajanian A (1998). Family and family change in Iran. Diversity in families: A global perspective.

[29] Farahani MA, Sahragard R, Carroll JK, Mohammadi E (2011). Communication barriers to patient education in cardiac inpatient care: A qualitative study of multiple perspectives. International Journal of Nursing Practice.

[30] Sirois FM (2008). Motivations for consulting complementary and alternative medicine practitioners: a comparison of consumers from 1997-8 and 2005. BMC Complement Altern Med.

[31] Mazess RB (1968). Hot-Cold Food Beliefs Among Andean Peasants. Journal of the American Dietetic Association.

[32] Dickson A, O’Brien G, Ward R (2011). Adjustment and coping in spousal caregivers following a traumatic spinal cord injury: an interpretative phenomenological analysis. Journal of Health Psychology.

[33] Baldacchino D, Draper P (2001). Spiritual coping strategies: a review of the nursing research literature. Journal of Advanced Nursing.

[34] Folkman S, Chesney M, McKusick L, et al, Eckenrode J (1991). Translating coping theory into an intervention. The social context of coping.

